# Synergistic Effects of Zinc Oxide Nanoparticles and Bacteria Reduce Heavy Metals Toxicity in Rice (*Oryza sativa* L.) Plant

**DOI:** 10.3390/toxics9050113

**Published:** 2021-05-20

**Authors:** Nazneen Akhtar, Sehresh Khan, Shafiq Ur Rehman, Zia Ur Rehman, Amana Khatoon, Eui Shik Rha, Muhammad Jamil

**Affiliations:** 1 Department of Biotechnology and Genetic Engineering, Kohat University of Science & Technology (KUST), Kohat 26000, Pakistan; nazneen_kht92@yahoo.com (N.A.); sehreshkhan91@yahoo.com (S.K.); ziamarwat77@gmail.com (Z.U.R.); 2 Department of Biology, University of Haripur, Haripur 22620, Pakistan; drshafiq@yahoo.com; 3 Department of Environmental and Botanical Sciences, Kohat University of Science & Technology (KUST), Kohat 26000, Pakistan; proteomics.sp@gmail.com; 4 Department of Well-Being Resources, Sunchon National University, Suncheon 540-742, Korea

**Keywords:** heavy metals, polluted water, synergistic, bacteria, nanoparticles

## Abstract

Heavy metals (HMs) are toxic elements which contaminate the water bodies in developing countries because of their excessive discharge from industrial zones. Rice (*Oryza sativa* L) crops are submerged for a longer period of time in water, so irrigation with HMs polluted water possesses toxic effects on plant growth. This study was initiated to observe the synergistic effect of bacteria (*Bacillus cereus* and *Lysinibacillus macroides)* and zinc oxide nanoparticles (ZnO NPs) (5, 10, 15, 20 and 25 mg/L) on the rice that were grown in HMs contaminated water. Current findings have revealed that bacteria, along with ZnO NPs at lower concentration, showed maximum removal of HMs from polluted water at pH 8 (90 min) as compared with higher concentrations. Seeds primed with bacteria grown in HM polluted water containing ZnO NPs (5 mg/L) showed reduced uptake of HMs in root, shoot and leaf, thus resulting in increased plant growth. Furthermore, their combined effects also reduced the bioaccumulation index and metallothionine (MTs) content and enhanced the tolerance index of plants. This study suggested that synergistic treatment of bacteria with lower concentrations of ZnO NPs helped plants to reduce heavy metal toxicity, especially Pb and Cu, and enhanced plant growth.

## 1. Introduction

Rapid increase in industrialization and anthropogenic activities has greatly contributed towards Heavy Metals (HMs) pollution in water which affects all aspects of the ecosystem [1]. Heavy metals are metals with relatively high density that are toxic in small (e.g., Cd, Pb, Hg, As) or higher (e.g., Cu, Zn, Co) concentrations [2]. In Pakistan, no sewage treatment plants are available, so crop irrigation with HM contaminated water results in metal accumulation in grains and other tissues of the plant [3]. It is evident from the literature [4] that water samples obtained from Hayatabad Industrial Estate (HIE), Peshawar, Pakistan, showed a higher level of HM contamination than quality standards should allow. Rice is the main staple food used by 2.7 billion people globally as a daily energy requirement. In Pakistan, its production is badly affected due to excesses of HM contamination in irrigated water [5]. Basically, HMs affect the biological systems of rice plants by altering several processes such as water, nutrients and oxygen uptake, and damage the chlorophyll synthesis pathway [6]. HMs damage the structure and quantity of seeds, the area of sugars grains, the cellular organelles, and also break the mitochondria, the nuclear envelope and damage the wall thickness of the plant cell [7]. Toxic metals disturb osmoregulation during the development process in plants by increasing osmolarity, reducing water potential, affecting the leaf area and transpiration level, and creating reactive oxygen species (ROS) by oxidative burst of the lipid membrane, protein, and DNA [8]. Plants protect themselves from the toxic effect of HMs by binding with metal binding proteins (metallothionein), and sequester it inside the vacuole [9]. Metallothionein (MTs) stress biomarkers in plants which are expressed during stressful (salinity, drought, temperature and heavy metal stress) environments [9]. In the past, various approaches were used to remediate HMs from water, such as ion exchange, precipitation, adsorption, reverse osmosis, and sedimentation, but these are more expensive and time-consuming techniques than biological methods [10]. Currently, two approaches are commonly used to remediate HMs in water: bioremediation and nano-remediation [11]. Bacteria remediate HMs from water by using specific detoxifying mechanism such as bio-transformation, bio-accumulation and bio-precipitation [12]. However, the solubilized and dispersed form of heavy metals in water cannot be easily and efficiently removed by bacteria [13]. Therefore, there was a great demand to use adsorbents with effective binding capability to heavy metals, which can subsequently help in remediation of HMs in wastewater. ZnO NPs are environmentally friendly adsorbents for the removal of HMs from water due to the different functional groups on the surface, which are exchanged with HM ions in water solutions and improved plant growth [14]. ZnO NPs interact with HMs by adopting the adsorption mechanism (at neutral PH, the surface of ZnO NPs was negative, thus increasing electrostatic interaction between NPs and metals ions) and breaking the water molecules into hydrogen and oxygen, which further change into superoxide O^2−^ and bind with H^+^ to form HO_2_ radical [15]. At higher concentrations, these radicals react with H^+^ ions to produce H_2_O_2_, into the cell membrane, and can kill the bacteria [12,13,14,15,16]. Zinc oxide NPs at lower concentrations (5 and 10 mg/L) are essential constituents for the growth of bacteria and have a positive effect on enzymes such as dehydrogenase, thiol peroxidase and glutathione reductase, which increase the resistance level of bacteria against HMs [17].

Bacteria also play a significant role in the enhancement of the phytoremediation potential of plants; e.g., bacterial-priming enhances the germination of seeds by utilizing minerals such as iron, phosphate, and nitrogen, preventing the plant from different diseases [18]. Seed priming with the *Bacillus* species improved seed germination because of the secretion of amino cyclopropane (1-carboxylic acid (ACC) deaminase) enzymes that work as growth hormones [10]. *Bacillus* species promote seed growth via two types of mechanism: firstly, increasing phosphorus uptakes and, secondly, by biosynthesis of phytohormones (auxins (IAA), cytokines, and gibberellins), surfactant, fencing, and lipo-peptides that protect the seeds from abiotic stress [19]. Furthermore, ZnO NPs at lower concentration have a significant positive effect on plant growth and act as fertilizer, having less solubility and less toxicity towards microbes and plants than at higher concentrations [20], dispersing and releasing Zn^2+^ ions (positive charge ions) which have a high binding affinity toward HMs [21]. A higher concentration of ZnO NPs causes cell membrane disruption and induces intracellular oxidative species to include hydrogen peroxide and toxic agents, which are harmful to bacteria [22]. A recent report showed that ZnO NPs and titanium oxide nanoparticles efficiently remediate toxic metals from soil and increase the photosynthesis and metabolic activity of *Picochlorum* spp. plants [13].

It was reported [23] that bacteria remediate HMs from polluted water, but to the best of our knowledge, there is no data available on the combined application of bacteria and ZnO NPs to enhance the remediation potential of plants against HMs. Therefore, it was hypothesized that the synergistic treatments of bacteria and nanoparticles may significantly improve the remediating potential of plants in the toxic environment. The current study investigates the significance of combined treatments of bacteria and nano-particles on plants raised from seeds primed with *Bacillus* spp. and grown in HM contaminated water, along with a lower concentration (5 and 10 mg/L) of ZnO NPs, by using physiological, biochemical and heavy metal remediation, e.g., bioaccumulation and tolerance index methods.

## 2. Materials and Methods

### 2.1. Sampling

Wastewater sample was collected from the Hayatabad industrial estate, Peshawar, Pakistan in clean and dry plastic bottles and placed at 4 °C in the Lab. ZnO NPs were characterized by Amara et al. [24] through Field Emission Scanning Electron Microscope (FESEM), Transmission Electron Microscopy (TEM), Fourier Transform Infrared Spectroscopy (FTIR), and X-ray diffraction (XRD) and obtained from Pir Mehr Ali Shah, Arid Agriculture University, Rawalpindi, Pakistan. The stock solution of ZnO NPs was prepared by dissolving 1 g of ZnO NPs in 1000 mL of sterilized (Milli-Q water) (Sigma-Aldrich, St. Louis, CA, USA). In order to break up large aggregates of NPs and obtain homogenized solutions, the ZnO NPs solution was placed in an ultrasonic bath for 30 min. NPs were further sonicated for 30 min before each experiment.

### 2.2. Bacterial Strains Collection and Cultivation

The two HMs-resistant bacteria species *(Bacillus cereus* (PMBL-3) and *Lysinibacillus macroides* (PMBL-7), isolated from the industrial estate’s effluent at Gadoon, were identified by Khattak et al. [25]. These two strains were obtained from the Plant and Microbial Biotechnology Laboratory, Kohat University of Science and Technology, Kohat, Pakistan. Bacterial strains were grown on nutrient broth (1% tryptone (Sigma Aldrich, St. Louis, CA, USA) 1% sodium chloride (Sigma Aldrich, St. Louis, CA, USA), and 0.5% yeast extract (Sigma Aldrich, St. Louis, CA, USA) at pH 7.4–7.6. Fresh culture of the bacteria was inoculated in autoclaved media for 24 h. Afterwards, the culture broth containing bacteria was centrifuged at 3500× *g* for 10 min, and then the pellet was properly washed with 1 mL of 5% NaCl solution (Sigma Aldrich, St. Louis, CA, USA). The bacterial cells were suspended in sterilized saline water for further experiments.

### 2.3. Heavy Metals Analysis in Polluted Water Samples

Heavy metals (Pb, Cd, Cr, and Cu) content in polluted water was determined by following the methodology of Radulescu et al. [26]. HMs content was analyzed by adding 2 mL of 70% concentrated nitric acid (HNO_3_) (Sigma Aldrich, St. Louis, CA, USA) and 5 mL of 50% concentrated hydrochloric acid (HCl) (Sigma Aldrich, St. Louis, CA, USA) in (100 mL) water samples boiled at 95 °C on a hot plate (Ceramic hot plate C-MAG HP 4, Rawalpindi, Pakistan). Water was heated until the volume reduced to 15–20 mL. HMs content was than analyzed by atomic absorption spectroscopy (Perkin Elmer, Analyst 4000, Waltham, MA, USA).

### 2.4. Heavy Metals Remediation Analysis/Batch Culture Experiment

#### 2.4.1. Heavy Metals Remediation in Artificially Polluted Water

HMs (Pb, Cd, Cr, and Cu) remediation of artificially polluted water (100 mg/L of each metal) was carried out following the methodology of Bestawy [27]. Erlenmeyer flasks (250 mL) were filled with 100 mL of fresh bacteria strains culture (*B. cereus* and *L*. *macroides*), HMs (Pb, Cd, Cr, and Cu) solution and different concentrations of ZnO NPs (5, 10, 15, 20 and 25 mg/L). Flasks were regularly agitated at 200 rpm for 90 min in the shaker incubator until reaching equilibrium. Flasks were then centrifuged at 4000× *g* to separate the solid–liquid phase and the samples were filtered through a 0.22 µm filter membrane. The experiment consisted of one positive control (bacteria culture without ZnO NPs) and one negative control (only ZnO NPs). All the experiments were performed in triplicate. HM concentrations in filtered water samples were determined by atomic absorption spectroscopy. HM removal efficiency (%) was determined by the given formula [28]:

Removal Efficiencies (%) = (C_0_ − C_e_) × 100 (1)

C_0_ is the initial while C_e_ is the final concentration of HMs in solution.

#### 2.4.2. Heavy Metals Remediation of Hayatabad Industrial Estate (HIE) Polluted Water

The bio-nano remediation experiment further analyzed a polluted water sample designed in batch culture flasks by following the protocol of Bestawy [27] with slight modification. In this method, a series of 500 mL Erlenmeyer flasks were filled with 100 mL of polluted water with an equal volume (100 mL) of different concentrations of ZnO NPs (0, 5, 10, 15, 20 and 25 mg/L). Then 100 mL of fresh culture broth of *B. cereus* and *L. macroides* was added and agitated in incubator shaker at 200 rpm (90 min) until equilibrium was reached. The solution was centrifuged and the supernatant was used to investigate the HM (Pb, Cd, Cr and Cu) contents by atomic absorption spectroscopy.

#### 2.4.3. Heavy Metals Remediation at Different pH and Contact Time

Effect of pH and contact time on the removal efficiency of HMs ions were determined by the following methodology [29]. Effect of pH was determined by mixing 100 mL of fresh cultured broth of bacteria strains (*B*. *cereus* and *L. macroides*) with 100 mL of metal ions (Sigma Aldrich, St. Louis, CA, USA) (Pb, Cd, Cr and Cu) (100 mg/L) solution and 100 mL of different concentrations of ZnO NPs (5, 10, 15, 20 and 25 mg/L) solutions. All the flasks were agitated in a shaking incubator for 90 min at different pH (4, 5, 6. 7 and 8) conditions. The pH of each solution was maintained by adding 1 mL of 5 M hydrochloric acid (HCl) and 1 mL of 5 M sodium hydroxide (Sigma Aldrich, St. Louis, CA, USA) (NaOH). The pH of each flask was determined by pH meter (OAKIAN digital pH meter, Waltham, MA, USA). Further experiments were carried out at different time intervals (30, 60, 90, and 120 min) while pH remained the same. Time at each interval was determined by Time clock (Time clipart digital clock, Waltham, MA, USA).

### 2.5. Heavy Metals Remediation in Plants

The remediation potential of the combined treatment of bacteria and ZnO NPs was further observed in the hydroponic culture experiment. Seeds were primed with bacterial strains (*B. cereus* and *L. macroides*) and grown in distilled water for 10 days. After 10 days, young seedlings were immediately transplanted in trays (3 L) containing one quarter strength Hoagland solution. The Hoagland medium composition includes 2.5 mL of 2 M KNO_3_, 2 mL of 2 M Ca(NO_3_)_2_ × 4 H_2_O, 1.5 mL of Iron oxide, 1 mL of 2 M MgSO_4_× 7 H_2_O, 1 mL of 1 M KH_2_PO_4,_ and 1 mL micronutrients. Plants were grown in the green house at 30 ± 2 °C in 16 h light with 60% humidity. After 21 days of cultivation, seedlings were transferred from the Hoagland solution to the solutions containing 5 and 10 mg/L ZnO NPs in (1 L) and HM contaminated wastewater for 1 week (7 days). The hydroponic system was used to inhibit the sorption of ZnO NPs to the soil surface and ensure that NPs and HMs were fully available [21]. Three replicates for each treatment and control blank were grown. During the exposure time, plants were randomly rotated and relocated to ensure equal light exposure. After HM polluted water and ZnO NPs exposure, the pH of the growth medium was maintained with a pH meter every daily.

#### 2.5.1. Heavy Metals in Water and Plant Tissue (Root, Shoot and Leaf) after Remediation

HM content in different tissues of the plant was investigated by following the protocol of Retka [30]. Plant tissues (root, shoot and leaves) were dried at 80 °C in a dry oven (Dry heat Oven) for 2 days. Dried plant tissues (0.5 g) were taken and crushed with mortar and pestle separately. Crushed root, shoot, and leaf were digested with 3 mL of 70% (*v*/*v*) nitric acid and incubated overnight at room temperature. The next day, samples were heated at 95 °C for 4 h. After cooling, 2 mL of 30% (*v*/*v*) H_2_O_2_ was added in the beaker and reheated at 95 °C until samples were fully digested. HM contents in different plant tissues were analyzed by atomic absorption spectroscopy (Perkin, ELMER, and Analyst 4000).

#### 2.5.2. Heavy Metals Analysis in Polluted Water after Remediation Experiment

Heavy metals (Pb, Cd, Cr and Cu) content in polluted water was again analyzed by using the protocol of Radulescu [26]. To determine the HM content in treated water samples, 2 mL of concentrated nitric acid (HNO_3_) and 5 mL of hydrochloric acid (HCl) were added to the beaker. The solution was boiled on a hot plate (Ceramic hot plate C-MAG HP 4, Rawalpindi, Punjab, Pakistan) at 95 °C until volume decreased to 15–20 mL. HMs were determined by atomic absorption spectroscopy (Perkin Elmer, Analyst 4000, Waltham, MA, USA).

### 2.6. Quantification of Low Molecular Weight Polypeptide Metallothioneins (MTs)

Metallothionein content was quantified through spectroscopy by following the protocol as described previously by Palmiter et al. [31]. Plant samples were homogenized in a buffer solution containing 5 M sucrose (Sigma-Aldrich, St. Louis, CA, USA), 20 mM Tris-HCl (Sigma-Aldrich, St. Louis, CA, USA) (pH 8.6) and 0.01% of β-mercapto-ethanol (Sigma-Aldrich, St. Louis, CA, USA). The mixtures were vortexed and spun at 10,000 rpm for 30 min. Clear supernatant was taken and 1 mL chilled ethanol (Sigma Aldrich, St. Louis, CA, USA) was added. After 5 min, supernatant was mixed with 80 μL chloroform (Sigma Aldrich, St. Louis, CA, USA). The solution was again spun in a micro-centrifuge at 6000 rpm for 10 min (Micro centrifuge RM-03 plus, Waltham, MA, USA). Subsequently, 1 mL of cold 70% ethanol (ethyl) was mixed with supernatant and heated at room temperature (RT) for 1 h until the pellet was formed. Afterwards, a buffer solution (ethanol:chloroform in 87:12 *v*/*v* ratio) was added into the pellet. Furthermore, 100 μL of 5 mM Tris-HCl and 1 mM of ethylene diamine tetra-acetic acid (EDTA) (pH 7) were added to the resulting pellet. In the resulting crude mixture of metallothioneins, 40 μL of 0.43 mM of nitrobenzoic-acid (pH 8) and 5 μL of 2 M of phosphate buffer (PBS) was added for 30 min and the absorbance recorded at 412 nm. The MTs activity was measured by the estimated curve of glutathione (GSH) with the following equation, assuming that 1 mol of MT contains 20 mol of cystein [31]:

Total glutathione (GSSG + GSH) = [∆OD at 415/min − (y-intercept)] × sample dilution × 2 *(2)

* The equation is multiplied by 2 as 1 GSSG= 2 GSH.

Metallothionein (MT) protein quantity was measured by the methodology of Tsugama et al. [32]. Plant material was boiled in 0.5 M protein extraction buffer (Sigma-Aldrich, St. Louis, CA, USA) (1 mM EDTA (pH 8), 1 M of Tris-HCl (pH, 6.8), 10% *w*/*v* SDS (Sigma-Aldrich, St. Louis, CA, USA), 100% β-mercapto-ethanol and 80% glycerol (Sigma-Aldrich, St. Louis, CA, USA) with a small amount of bromophenol blue (Sigma-Aldrich, St. Louis, USA). The solution was maintained at pH 7.4 and placed for 5–10 min. Protein was estimated through Bradford’s method by following the protocol as described previously by Laemmli [33]. The low molecular weight proteins of MTs were separated on a 17% SDS twin mini gel electrophoresis unit (Bio-Rad, Hercules, CA, USA) at 80 V for 2.5 h. The gel was stained for 20 min by adding Coomassie blue dye (R-250, Sigma-Aldrich, St. Louis, CA, USA) and 20% methanol. The sample was de-stained by adding 5% acetic acid (Sigma-Aldrich, St. Louis, CA, USA), and then bands were compared with standard protein markers (Benchmark Protein Ladder, Thermo Fisher Scientific Inc. Waltham, MA USA) in the electro photogram (Bio-Rad, Hercules, CA, USA).

### 2.7. Bioaccumulation Index and Tolerance Index (TI) Determination

The bioaccumulation index was analyzed by measuring the number of heavy metals accumulated in grown plants following the previous method [34] with slight modification. The bioaccumulation index was calculated by using the following formula:

Bioaccumulation index (µg/g) = C^plant^/C^water^ (3)

C^plant^= Concentration of HMs in rice plant

C^water^= Concentration of HMs in water

The tolerance index of plants was measured by using the protocol of Wu et al. [35] with slight modification. The following formula was used to estimate the tolerance index (%) with slight modification.

TI (%) = (Dry weight of treated plants/Dry weight of control plants × 100)(4)

### 2.8. Statistical Analysis

The data were subjected to one-way ANOVA by using statistix 9 software (v.10, Informer Technologies, Inc., Los Angeles, CA, USA). Means were separated by following the least significance difference (LSD) at *p* ≤ 0.05.

## 3. Results

### 3.1. ZnO NPs Interaction with Bacteria Improved the Removal Efficiency of HMs

**Figure 1:**
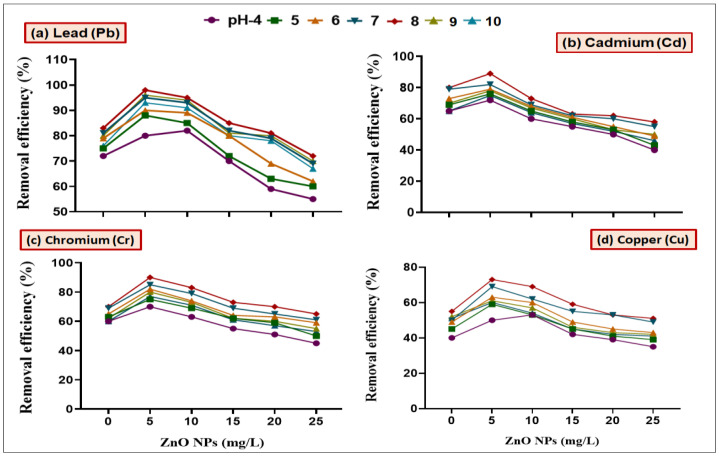
Synergistic effect of *B. cereus* and ZnO NPs (5, 10, 15, 20 and 25 mg/L) on the removal efficiency of (**a**) Lead (Pb), (**b**) Cadmium (Cd), (**c**) Chromium (Cr) and (**d**) Copper from the media amended with heavy metal at different pH (4, 5, 6, 7, 8, 9 and 10).

**Figure 2:**
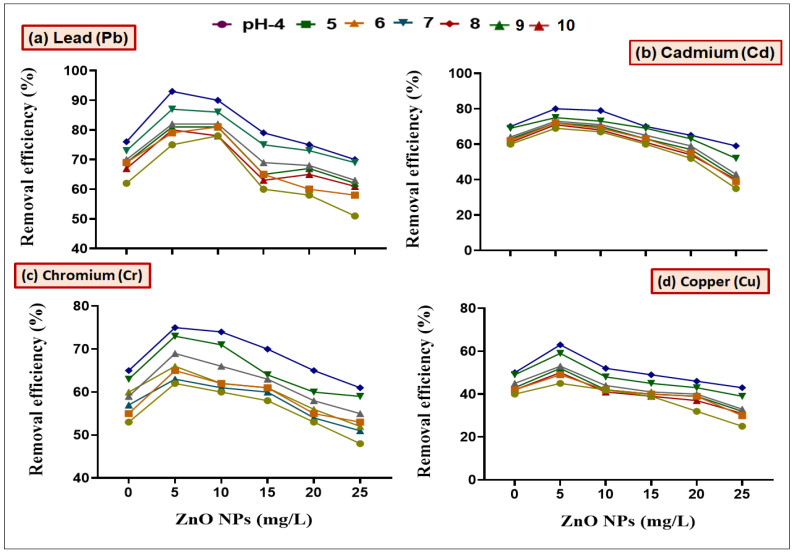
Synergistic effect of *L. macroides* and ZnO NPs (5, 10, 15, 20 and 25 mg/L) on the removal efficiency of (**a**) Lead (Pb), (**b**) Cadmium (Cd), (**c**) Chromium (Cr) and (**d**) Copper from the media amended with heavy metal at different pH (4, 5, 6, 7, 8, 9 and 10).

**Figure 3:**
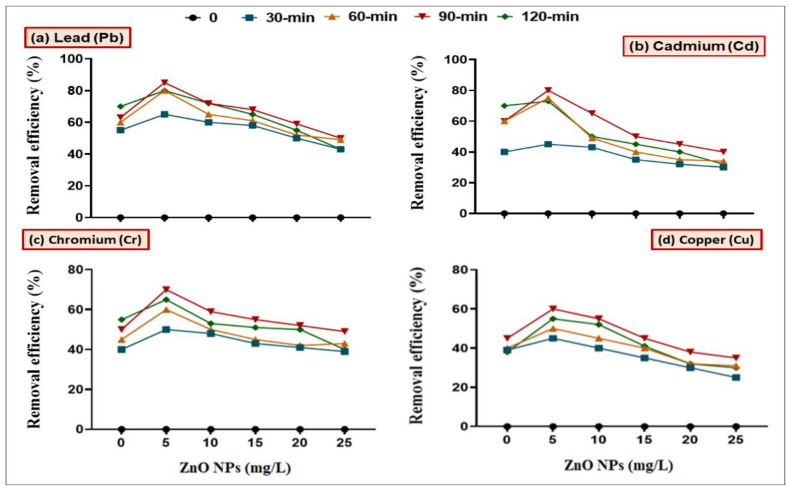
Synergistic effect of *B. cereus* and ZnO NPs (5, 10, 15, 20 and 25 mg/L) on the removal efficiency of (**a**) Lead (Pb), (**b**) Cadmium (Cd), (**c**) Chromium (Cr) and (**d**) Copper from the media amended with heavy metal at different time intervals (0, 30, 60, 90 and 120 min).

**Figure 4:**
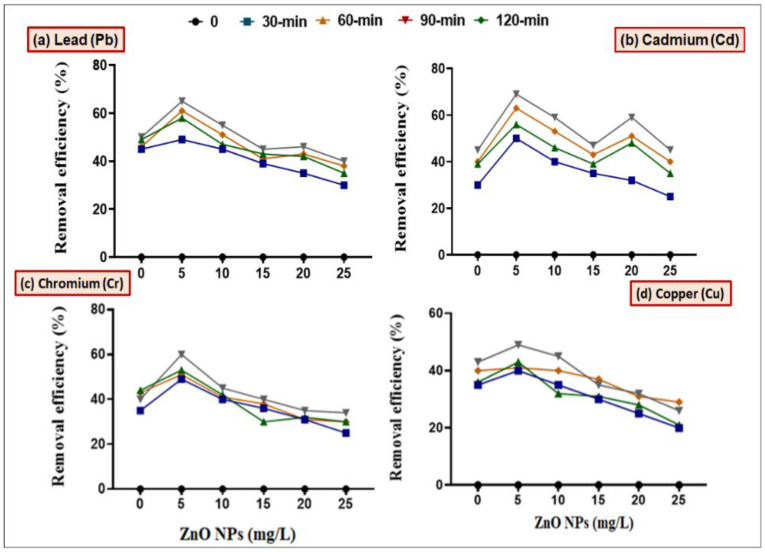
Synergistic effect of *Lysinibacillus macrolides* and ZnO NPs (5, 10, 15, 20 and 25 mg/L) on the removal efficiency of (**a**) Lead (Pb), (**b**) Cadmium (Cd), (**c**) Chromium (Cr) and (**d**) Copper from the media amended with heavy metal at different time intervals (0, 30, 60, 90 and 120 min).

In order to check the synergistic effect of ZnO NPs along with bacterial strains (*B. cereus* and *L. macroides*) in remediating HMs, we first wanted to determine the potential effect(s) of ZnO NPs on bacteria at different pH (4, 5, 6, 7, 8, 9 and 10) and contact time (0, 30, 60, 90 and 120 min) in batch culture experiments (Figure 1). Results revealed that maximum remediation of Pb, Cd, Cr, and Cu was observed at lower concentration (5 mg/L) of ZnO NPs with *B. cereus* (98, 89, 90 and 73%) and *L. macroides* (93, 80, 73 and 59%) at pH 8, as compared with individual treatments of *B. cereus* (83 and 70%) and *L. macroides* (60 and 65%), respectively (Figure 2). Remediation efficiency was further observed at different time intervals (0, 30, 60, 90 and 120 min). Maximum removal efficiency was observed at 90 min time interval as compared with other conditions. Results revealed that the synergistic effect of *B. cereus* with 5 mg/L ZnO NPs showed maximum remediation efficiency (85 and 80, 70 and 60%) of Pb, Cd, Cr, and Cu as compared with individual treatments of *B. cereus* (80 and 60%) and *L. macroides* (55 and 50%), respectively (Figure 3 and Figure 4).

### 3.2. ZnO NPs Interaction with Bacteria Improved the Removal Efficiency of HMs from Polluted Water

We had observed that ZnO NPs at lower concentrations, along with bacteria, efficiently remediate HMs from the media amended with heavy metals at neutral pH (90 min). We further determined the effect of ZnO NPs at lower concentrations along with bacteria on polluted water. For this purpose, both bacterial strains (*B. cereus* and *L. macroides)* along with NPs were applied on HM contaminated water. Results revealed that maximum HMs (Pb (0.24 mg/L), Cd (1.40 mg/L), Cr (1.26 mg/L) and Cu (2.02 mg/L) contents were observed in non-treated polluted water. It was observed that *B. cereus* along with lower concentrations (10 mg/L) of ZnO NPs revealed a maximum reduction (0.12, 1.25, 1.15 and 1.08 mg/L) of HMs (Pb, Cd, Cr and Cu mg/L). On the contrary, higher concentrations (25 mg/L) of ZnO NPs with *B. cereus* (0.18, 1.29, 1.22 and 1.29 mg/L) and *L. macroides* (0.20, 1.32, 1.23 and 1.21 mg/L) showed a non-significant effect in HM (Pb, Cd, Cr and Cu) remediation (Table 1).

### 3.3. ZnO NPs Interaction with Bacteria Reduced HMs Uptake in Plant Tissues

#### 3.3.1. Heavy Metals Contents in Plant Tissue (Leaf, Shoot and Root)

**Figure 5:**
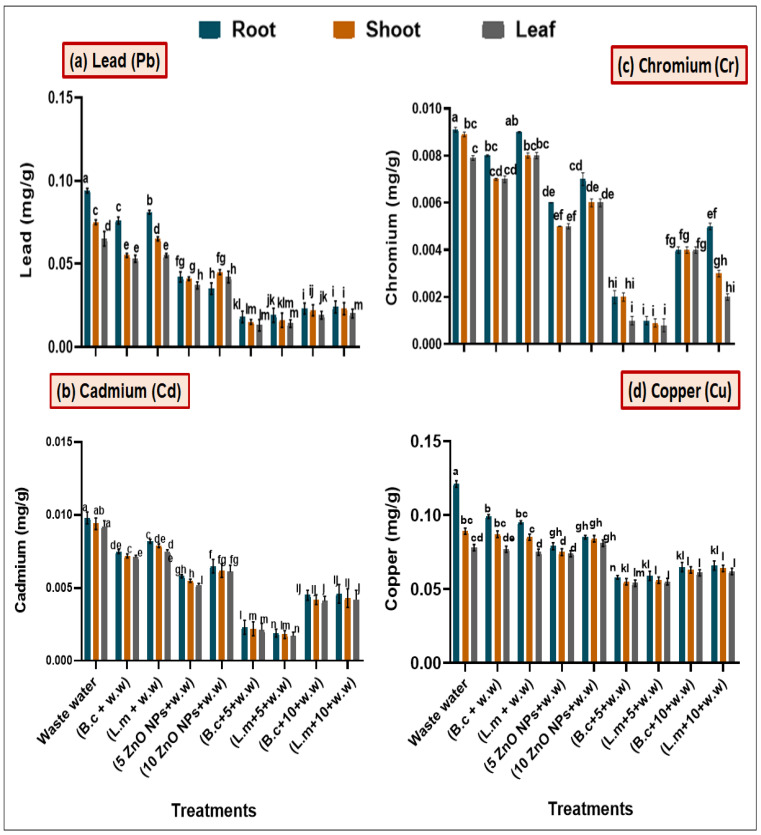
Synergistic effect of the bacterial strains (*B. cereus* and *L. macroides)* and ZnO NPs (5 and 10 mg/L) on heavy metals (**a**) Lead (Pb), (**b**) Cadmium (Cd), (**c**) Chromium (Cr) and (**d**) Copper (Cu) contents in root, shoot and leaf of rice grown in heavy metal contaminated water. Error bars show means of standard error (±S.E.) of three replicates (*n* = 3). Different alphabets appeared in superscript on each number showed statistically significant at 5% probability level.

Maximum accumulation of Pb, Cd, Cr, and Cu was observed in plants grown in HM polluted, as compared with combined (Bacteria-ZnO NPs), treated plants. Maximum Pb content was observed in leaf, shoot and root (0.065, 0.075 and 0.093 mg/g) in plants grown in HM contaminated water while plant grown in combined treatments of *B. cereus* (0.013, 0.014 and 0.017 mg/g) and *L. macroides* (0.014, 0.015 and 0.018 mg/g) at 5 mg/L ZnO NPs showed less accumulation of HMs as compared with individual treatment; *B. cereus* (0.053, 0.054 and 0.075 mg/g) and *L. macroides* (0.055, 0.064 and 0.080 mg/g) without ZnO NPs respectively (Figure 5). Cr content was maximum in leaf, shoot and root (0.0069, 0.0075 and 0.0091 mg/g) in plants grown in HM polluted water. Plant raised from seeds primed with *B. cereus* (0.006, 0.0065 and 0.007 mg/g) and *L. macroides* (0.007, 0.007 and 0.008 mg/g) grown in 5 mg/L ZnO NPs-containing polluted water showed less Cr content as compared with individual treatments; *B. cereus* (0.001, 0.002 and 0.008 mg/g) and *L. macroides* (0.006, 0.008 and 0.009 mg/g) without ZnO NPs treatments (Figure 5).

Results also revealed that Cd content was high in plant tissue (leaf, shoot, and root) (0.0090, 0.0093 and 0.0097 mg/g) grown in HM polluted water. Plants primed with *B. cereus* (0.0021, 0.0022, 0.0024 mg/g) and *L. macroides* (0.0016, 0.0017 and 0.0018 mg/g) grown in polluted water containing 5 mg/L ZnO NPs showed less Cd content as compared with individual plants raised from seeds primed with *B. cereus* (0.0070, 0.0071 and 0.0074 mg/g) and *L. macroides* (0.0074, 0.0078 and 0.008 mg/g) without ZnO NPs treatments (Figure 5). Cu content was high in leaf, shoot and root (0.075, 0.08 and 0.121 mg/g) of plant grown in HM polluted water. Plants raised from seeds primed with *B. cereus* (0.0049, 0.053 and 0.054 mg/g) and *L. macroides* (0.054, 0.055 and 0.057 mg/g) grown in polluted water containing 5 mg/L ZnO NPs showed less Cu content as compared with plants primed with *B. cereus* (0.076, 0.086 and 0.089 mg/g) and *L. macroides* (0.074, 0.084 and 0.09 mg/g) without ZnO NPs (Figure 5d).

#### 3.3.2. Total Heavy Metal Uptake, and Remediation Percentage in Polluted Water

Plants raised from bacterial primed seeds grown at the lower concentration of ZnO NPs revealed a lower uptake of HM content as compared with control treatments (Table 2). Maximum uptake of HMs (Pb, Cd, Cr and Cu) was observed in plants grown in HM contaminated water (0.211, 0.028, 0.025 and 0.014 mg/g). Synergistic treatment of seeds priming with *B. cereus* (0.046, 0.006, 0.005 and 0.167 mg/g) and *L. macroides* (0.049, 0.005, 0.006 and 0.171 mg/g) and grown in polluted water containing 5 mg/L ZnO NPs revealed minimum uptake of HMs as compared with individual treatment of *B. cereus* (0.184, 0.021, 0.022 and 0.263 mg/g) and *L. macroides* (0.201, 0.023, 0.025 and 0.255 mg/g) (Table 2).

Water analysis revealed that bacteria-NP combined treatments significantly decreased the HM content in polluted water as compared with individual treatments (Table 2). Maximum uptake of HMs (Pb, Cd, Cr and Cu) was observed in polluted water (without treated) samples (1.645, 0.269, 0.161 and 0.498 mg/L);while HMs (Pb, Cd, Cr and Cu) content were lowered in a plant grown from seeds primed with *B. cereus* (1.321, 0.091, 0.057 and 0.321 mg/L) and *L. macroides* (1.351, 0.121, 0.059 and 0.356 mg/L) in polluted water containing 5 mg/L ZnO NPs as compared with individual treatments; *B. cereus* (1.623, 0.219, 0.154 and 0.465 mg/L) and *L. macroides* (1.633, 0.221, 0.156 and 0.470 mg/L) without ZnO NPs treatments respectively (Table 2).

Maximum remediation of HMs was observed in synergistic (bacteria along with 5 and 10 mg/L ZnO NPs) treated plants of seeds as compared with HM grown plants (9.3, 2.84, 2.59 and 8.3 mg/L) (Table 2). Maximum reduction of metals (Pb, Cd, Cr and Cu) was observed in the synergistic treatment of seeds primed with *B. cereus* (41.8, 20.8, 13 and 2.6) and *L. macroides* (38.5, 17.8, 12.8 and 22.5 mg/L) and grown in polluted water containing 5 mg/L ZnO NPs as compared with individual treatments of *B. cereus* (11.5, 7.9, 3.3 and 11.6) and *L. macroides* (10.5, 7.7, 3.1 and 10.5) (Table 2).

### 3.4. Concentrationof Low Molecular Weight Polypeptide Metallothioneins (MTs)

**Figure 6:**
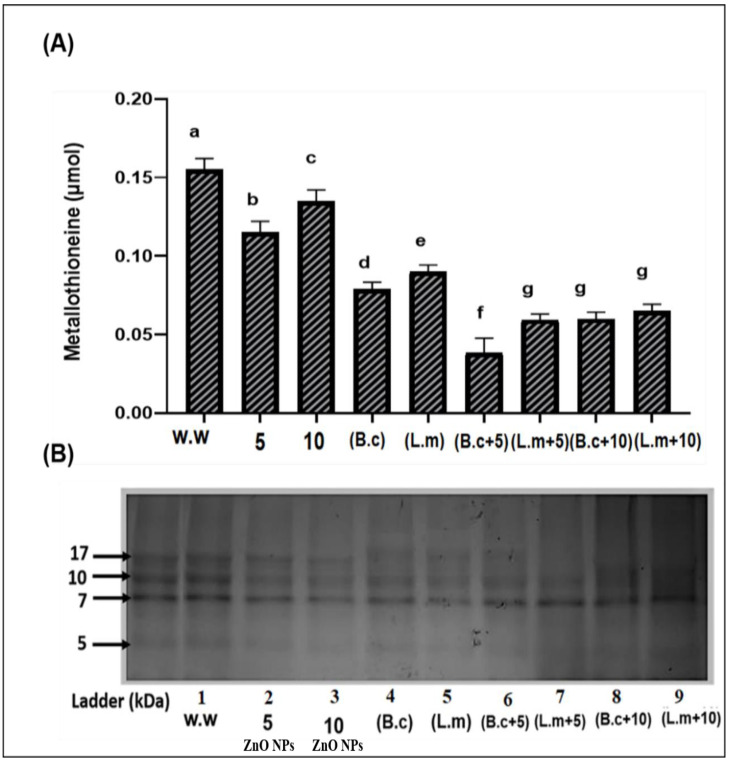
Synergistic effect of the bacterial strains (*B. cereus* and *L. macroides*) and ZnO NPs (5 and 10 mg/L) on Metallothioneins (MTs) contents (**A**) and concentration (**B**) of rice plants grown in HMs contaminated water. Lane 1: w.w (Waste water)., Lane 2: 5 mg/L ZnO NPs + w.w., Lane 3: 10 mg/L ZnO NPs + w.w., Lane 4: B.c +w.w, Lane 5: L.m + w.w., Lane 6: B.c. + 5 (*B. cereus* + 5 mg/L ZnO NPs + w.w.)., and Lane 7: L.m + 5 (*L. macroides* + 5 mg/L ZnONPs + w.w.), Lane 8: B.c. + 10 (*B. cereus* + 10 mg/L ZnO NPs +w.w.) and Lane 9: L.m. + 10 (*L. macroides* + 10 mg/L ZnO NPs + w.w.). Error bars show means of standard error (±S.E.) of three replicates (*n* = 3) showing statistical significance at 5% probability level (ANOVA). Different alphabets appeared in superscript on each number showed statistically significant at 5% probability level.

It is evident from the earlier section that ZnO NPs and bacteria combined treatments, at lower concentrations, improved plant growth under HM stress. In order to prove their role in proteomic level, we determined metallothioneins (MTs) content, as mentioned in materials and methods. It is clear from Figure 6 that the synergistic effect of bacterial strains and ZnO NPs showed minimum MTs content under HMs stress as compared to the individual treatments of bacteria and ZnO NPs. Plants grown in HM contaminated water showed maximum 0.155 µmol MTs content. On the contrary, plants raised from seeds primed with *B. cereus* and *L. macroides* grown in polluted water containing 5 mg/L ZnO NPs had the lowest MTs content (0.0385 and 0.059 µmol) compared with plants raised from primed seeds with *B. cereus* and *L. macroides* (0.1150 and 0.1350 µmol) (Figure 6A). MT concentration was also tested by using SDS PAGE. The synergistic treatment of *B. cereus* and *L. macroides* along with ZnO NPs at 5 mg/L in the presence of HMs showed a smaller number of bands at 7 kDa as compared with *B. cereus* and *L. macroides* primed seeds without ZnO NPs treatments and individually grown plants at 5 mg/L ZnO NPs treatments, respectively (Figure 6B).

### 3.5. Bioaccumulation Index

**Figure 7:**
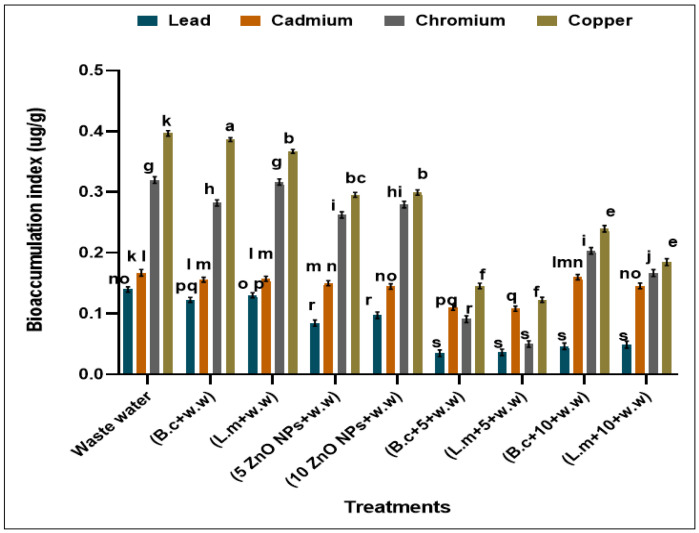
Bioaccumulation factor (BAF) of HMs in rice plants grown in HM polluted water. Waste water., B.c + w.w (*B. cereus* + waste water), L.m *+* w.w (*L. macroides* + waste water), 5 ZnO NPs + w.w (5 mg/L ZnO NPs + waste water), 10 ZnO NPs + w.w (10 mg/L ZnO NPs + waste water), B. c. + 5 + w.w (*B. cereus* + 5 mg/L ZnO NPs + waste water), L.m + 5 + w.w (*L. macroides* + 5 mg/L ZnONPs + waste water), B.c. + 10 + w.w (*B. cereus* + 10 mg/L ZnO NPs + waste water), L.m. + 10 + w.w (*L. macroides* + 10 mg/L ZnO NPs + waste water). Error bars show means of standard error (±S.E.) of three replicates (*n* = 3) showed statistical significance at 5% probability level (ANOVA). Different alphabets appeared in superscript on each number showed statistically significant at 5% probability level.

Bioaccumulation index decreased in the plants after synergistic treatment with bacteria and NPs as compared with HMs (Figure 7). Maximum accumulation of Pb, Cd, Cr, and Cu was observed in plants grown in HMs polluted water (0.139, 0.167, 0.319 and 0.522 µg/g) while minimum accumulation was observed in plants raised from seeds primed with *B. cereus* (0.034, 0.11, 0.090 and 0.347 µg/g) and *L. macroides* (0.36, 0.108, 0.05 and 0.352 µg/g) grown in polluted water contained 5 mg/L ZnO NPs as compared with individual treatments of *B. cereus* (0.122, 0.155, 0.282 and 0.486 µg/g) and *L. macroides* (0.130, 0.157, 0.316 and 0.467 µg/g) without ZnO NPs (Figure 7).

### 3.6. Tolerance Index (TI)

**Figure 8:**
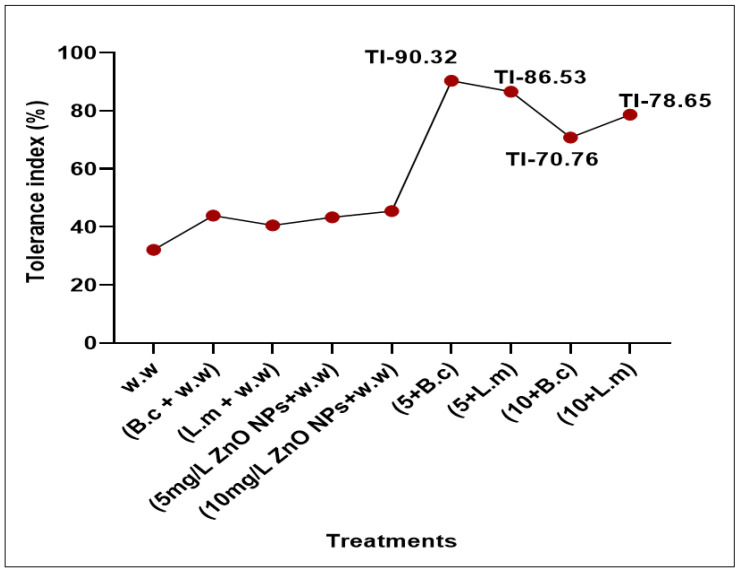
Tolerance index (TI) of rice plants grown in HM polluted water contained 5 and 10 mg/L ZnO NPs raised from seeds primed with bacterial strains (*B. cereus* and *L. macroides*). Waste water, B.c + w.w (*B. cereus* + waste water)., L.m *+* w.w (*L. macroides* + waste water)., 5 ZnO NPs + w.w (5 mg/L ZnO NPs + waste water), 10 ZnO NPs + w.w (10 mg/L ZnO NPs + waste water), B. c. + 5 + w.w (*B. cereus* + 5 mg/L ZnO NPs + waste water)., L.m + 5 + w.w (*L. macroides* + 5 mg/L ZnONPs + waste water), B.c. + 10 + w.w (*B. cereus* + 10 mg/L ZnO NPs + waste water), L.m. + 10 + w.w *(L. macroides* + 10 mg/L ZnO NPs + waste water).

The tolerance index was decreased in plants grown in polluted water (32.102%) while increased in plants grown after synergistic treatment with bacteria and NPs (Figure 8). However, as expected, maximum tolerance index was observed in the plants grown in polluted water containing 5 mg/L ZnO NPs raised from seeds primed with *B. cereus* (90.32%) and *L. macroides* (86.54%) as compared with individual treatments of *B. cereus* (43.86%) and *L. macroides* (40.52%) without ZnO NPs.

## 4. Discussion

Pakistan has an abundance of surface and groundwater resources, but due to industrial discharge, these resources are polluted with HMs, used by farmers for irrigation [36]. HMs also affect plant growth by inhibiting the cellular process, causing low pigmentation and crop production [37]. The individual effect of NPs and bacteria was observed for the remediation of HM contaminated water, but no work so far has been reported on the combined effect of *Bacillus* spp. and ZnO NPs on the remediation of HMs in rice plants. ZnO NPs have received great attention worldwide and caused detoxification and transformation of metals more efficiently in plants [38]. They remain suspended in water without electrostatic forces, so they provide significant tolerance against HMs [2].

It was observed from the current findings that the removal efficiency of combined treatment of bacteria and ZnO NPs (5 mg/L) was maximum at neutral pH (Figure 1 and Figure 2). This is in agreement with previous observations, where it has been shown that neutral pH condition made the surface of ZnO NPs more negative; thus electrostatic interactions between NPs and metal cations increased, which caused higher removal efficiency, while at low pH, HMs precipitate in the form of hydroxides where hydrogen ions compete for binding with adsorbents. These results were confirmed by the findings of Xie [39] that the hydroxide ions on the functional group of NPs react with hydrous oxide at higher pH to produce deprotonated oxide (MO^−^), while at lower pH, the hydrous surface will be completely covered with hydrogen ions [14]. The synergistic effect is more helpful in remediating the Pb and Cu metals from polluted water. It was reported by Karn [38] that ZnO nanoparticles showed 85% removal efficiency of Pb and Cu metal ions in the form of metal reduction/oxidation or adsorption mechanism. The removal efficiency of HMs was also observed at different time intervals (0, 30, 60, 90 and 120 min). Maximum removal efficiency of HMs was observed after a 90 min time interval (Figure 3 and Figure 4). It can be observed from the current findings that bacteria inoculation reduced the passage of metals in water, which results in low reactive oxygen species (ROS) in less time. These results were confirmed by the findings of Wang [40] that after 90 min maximum binding sites on NPs’ surface were exposed, which effectively bind with metals [41].

The batch culture experiment showed that lower concentrations (5 and 10 mg/L) of ZnO NPs improved the tolerance capacity of bacteria against heavy metals as compared with higher concentrations (Table 1). ZnO NPs at lower concentration (5 and 10 mg/L) help bacteria in remediating HMs in polluted water. *Bacillus* spp. are actively involved in the biotransformation of metals and use Zn^+2^ ions in the range of (0.01–1 mM) as micro-nutrients for their growth, stabilizing the membrane, macromolecules, different steroid receptors and carbohydrate metabolism under stress conditions. These findings revealed that zinc ions are beneficial for Zn regulatory proteins in bacteria, so increasing the tolerance capacity of bacteria against HMs [42]. It was reported by Ashraf [43] that the bacteria strain *Klebsiella variicola* isolated from industrial effluents has high bio-sorption ability and maximum tolerance against HMs. A recent report has been published by Wątły [44], in which it was observed that the application of titanium oxide NPs and ZnO NPs provided more active sites for HMs and increased remediation of metals.

In addition, it was observed that lower concentrations of ZnO NPs reduced the passage of HMs into different tissues of rice plants (Figure 5) in the presence of bacteria. Bio-priming of seeds with *Bacillus* species has been investigated to increase the nutritional pathway of rice under HM stress conditions [45]. Bacteria secreting plant growth-promoting metabolites extract nutrients from water by fixing nitrogen [46]. *Bacillus* spp. is the most notable genus used in abiotic stress tolerance in potato radish, rice, mung bean and chickpea [47]. The recent Amiard report [48] revealed that a significant reduction of HMs occurred in plants in two ways; the higher secretion of root exudates leads to higher adsorption of HMs on ZnO NPs; surface so reducing the bioavailability of metals. Low pH (5.4–5.8) is associated with retaining most ions at a free-standing position, while higher pH lowers the nutrient bioavailability. It was reported by Sharifan [49] that ZnO NPs act as bio-sorbent for the remediation of cobalt from water, used for the irrigation of crops.

Metallothionein (MTs) content in plants under HM stress is a well-known phenomenon [50]. Current research demonstrated that plants grown under HM stress had high MTs contents as compared with control plants (Figure 6). Our results showed resemblance to the findings of Khati et al. [51], who revealed that MTs content increased in plants under cadmium (Cd) stress. *B. cereus* and *L. macroides* primed seeds with ZnO NPs reduced HMs stress and lowered MT content. HM-resistant bacteria strains prevent the plant from a toxic effect and restrict their inflow in the plant. Earlier studies reported maximum MT contents in *lupinus luteus* L. plant under HMs stress [52].

Current findings also revealed that the number of HMs was reduced in water by bacteria-nanoparticles interaction (Table 2). It was also observed in the current study that bioaccumulation index HMs (Pb, Cd, Cr, and Cu) in the plant decreased and tolerance index increased in plants raised from bacteria primed seeds after application of NPs, as compared with HM polluted water (Figure 7). Metal reduction may be due to the ability of heavy metal-resistant bacteria to immobilize the HMs in the root by different mechanisms [52]. According to Abraham [46], seed inoculation with Cr resistance strain *P. aeruginosa* reduces the amount of chromium and increases plant growth. It was reported by Wu [35] that seed priming with bacteria increased the germination and tolerance capacity of crops by adhering to the seeds and decreased the passage of HMs to plants. It was also reported that ZnO NPs have a high surface to volume ratio in reacting with metals in contaminated media and also triggers the movement of metals in biochemical pathways in plants [44]. It was investigated by Dubchak [53] that metal oxide NPs such as Fe_3_O_4_, ZnO and CuO remove the metals from aqueous solution. These results were further confirmed by Kummar et al. [29], who observed that metal oxide NPs such as Fe_3_O_4_, ZnO and CuO removed the metals from aqueous solution and increased the germination of Mung (*Vignaradiata*) and gram (*Cicer aretinium*) plants.

## 5. Conclusions

It was concluded from the current study that ZnO NPs (at lower concentrations), along with bacteria, more efficiently bind with HMs ions and remediate metals from polluted water, as compared to their individual effect. The combined effect also showed low bioaccumulation index, metallothionine (MTs) content and inhibited the passage of HMs to plant tissues by improving the plant tolerance index. The combined treatments of ZnO NPs and bacteria play a significant role in plant tolerance level and the removal of HMs from water.

## Figures and Tables

**Table 1:** Synergistic effect of the bacterial strains (*B. cereus* and *L. macroides*) and ZnO NPs (5, 10, 15, 20 and 25 mg/L) on the polluted water sample collected from Hayatabad Industrial Estate (HIE), Pakistan.

Treatments	Lead (0.24 mg/L)		Cadmium (1.40 mg/L)		Chromium (1.26 mg/L))		Copper (2.02 mg/L)	
ZnO NPs	*B. cereus*	*L. macroides*	*B. cereus*	*L. macroides*	*B. cereus*	*L. macroides*	*B. cereus*	*L. macroides*
0 mg/L	0.20 + 0.1 ^ab^	0.21 + 0.3 ^ef^	1.33 + 0.2 ^ab^	1.35 + 0.3 ^bc^	1.23 + 0.2 ^cd^	1.19 + 0.3 ^fg^	1.18 + 0.21 ^bc^	1.17 + 0.21 ^c^
5 mg/L	0.11 + 0.2 ^bc^	0.14 + 0.4 ^fg^	1.22 + 0.3 ^cd^	1.25 + 0.1 ^cd^	1.11 + 0.3 ^fg^	1.10 + 0.4 ^ef^	1.11 + 0.21 ^cd^	1.12 + 0.21 ^cde^
10 mg/L	0.12 + 0.3 ^c^	0.15 + 0.2 ^gh^	1.25 + 0.1 ^cde^	1.26 + 0.2 ^cd^	1.15 + 0.4 ^efg^	1.16 + 0.3 ^gh^	1.08 + 0.21 ^cde^	1.09 + 0.21 ^cd^
15 mg/L	0.15 + 0.1 ^cd^	0.18 + 0.1 ^efg^	1.27 + 0.2 ^cd^	1.28 + 0.3 ^c^	1.17 + 0.5 ^bcd^	1.18 + 0.2 ^fg^	1.12 + 0.21 ^bc^	1.13 + 0.21 ^bcd^
20 mg/L	0.18 + 0.2 ^bc^	0.19 + 0.2 ^fg^	1.29 + 0.3 ^ab^	1.30 + 0.4 ^bc^	1.21 + 0.3 ^bc^	1.22 + 0.1 ^c^	1.15 + 0.21 ^a^	1.16 + 0.21 ^abc^
25 mg/L	0.18 + 0.1 ^abc^	0.20 + 0.3 ^cd^	1.29 + 0.4 ^ac^	1.32 + 0.2 ^ab^	1.22 + 0.3 ^ab^	1.23 + 0.2 ^cd^	1.19 + 0.21 ^ab^	1.21 + 0.21 ^ab^

Different alphabets appeared in superscript on each number showed statistically significant at 5% probability level.

**Table 2:** Synergistic effects of the bacterial strains (*Bacillus cereus* and *Lysinibacillus macroides)* and ZnO NPs (5 and 10 mg/L) on heavy metal uptake (mg/L) and reduction (%) of rice seedling grown in HM polluted water.

Treatments	Lead (1.738 mg/L)			Cadmium (0.298 mg/L)			Chromium (0.187 mg/L)			Copper (0.581 mg/L)		
	Water Analysis (mg/L)	Plant Uptake (mg/L)	Remediation (%)	Water Analysis (mg/L)	Plant Uptake (mg/L)	Remediation (%)	Water Analysis (mg/L)	Plant Uptake (mg/L)	Remediation (%)	Water Analysis (mg/L)	Plant Uptake (mg/L)	Remediation (%)
Polluted water	1.645	0.234	9.3	0.296	0.028	2.84	0.161	0.025	2.59	0.498	0.288	8.3
*Bacillus cereus (B.C.)*	1.623	0.184	11.5	0.219	0.0218	7.9	0.154	0.022	3.3	0.465	0.263	11.6
*Lysinibacillus macroides (L.M.)*	1.633	0.201	10.5	0.221	0.0236	7.7	0.156	0.025	3.1	0.476	0.255	10.5
5 mg/L ZnO NPs	1.564	0.12	17.4	0.165	0.0165	13.3	0.112	0.016	7.5	0.421	0.228	16
10 mg/L ZnO NPs	1.587	0.142	15.1	0.154	0.0188	14.4	0.123	0.019	6.4	0.425	0.25	15.6
(5 + *B.C.*)	1.32	0.046	41.8	0.09	0.0066	20.8	0.057	0.005	13	0.321	0.167	26
(5 + *L.M.*)	1.35	0.049	38.8	0.12	0.0054	17.8	0.059	0.0027	12.8	0.356	0.17	22.5
(10 + *B.C.)*	1.38	0.064	35.8	0.112	0.0128	18.6	0.078	0.012	10.9	0.376	0.189	20.5
(10 + *L.M.*)	1.39	0.068	34.8	0.125	0.0131	17.3	0.089	0.01	9.8	0.399	0.192	18.2

## Data Availability

Not applicable.
